# The Brain Emotional Systems in Addictions: From Attachment to Dominance/Submission Systems

**DOI:** 10.3389/fnhum.2020.609467

**Published:** 2021-01-15

**Authors:** Teodosio Giacolini, David Conversi, Antonio Alcaro

**Affiliations:** ^1^Department of Human Neuroscience, Sapienza University of Rome, Rome, Italy; ^2^Department of Psychology, Sapienza University of Rome, Rome, Italy

**Keywords:** addiction, adolescence, affective neuroscience, attachment, dominance/submission systems

## Abstract

Human development has become particularly complex during the evolution. In this complexity, adolescence is an extremely important developmental stage. Adolescence is characterized by biological and social changes that create the prerequisites to psychopathological problems, including both substance and non-substance addictive behaviors. Central to the dynamics of the biological changes during adolescence are the synergy between sexual and neurophysiological development, which activates the motivational/emotional systems of Dominance/Submission. The latter are characterized by the interaction between the sexual hormones, the dopaminergic system and the stress axis (HPA). The maturation of these motivational/emotional systems requires the integration with the phylogenetically more recent Attachment/CARE Systems, which primarily have governed the subject’s relationships until puberty. The integration of these systems is particularly complex in the human species, due to the evolution of the process of competition related to sexual selection: from a simple fight between two individuals (of the same genus and species) to a struggle for the acquisition of a position in rank and the competition between groups. The latter is an important evolutionary acquisition and believed to be the variable that has most contributed to enhancing the capacity for cooperation in the human species. The interaction between competition and cooperation, and between competition and attachment, characterizes the entire human relational and emotional structure and the unending work of integration to which the BrainMind is involved. The beginning of the integration of the aforementioned motivational/emotional systems is currently identified in the prepubertal period, during the juvenile stage, with the development of the Adrenarche—the so-called Adrenal Puberty. This latter stage is characterized by a low rate of release of androgens, the hormones released by the adrenal cortex, which activate the same behaviors as those observed in the PLAY system. The Adrenarche and the PLAY system are biological and functional prerequisites of adolescence, a period devoted to learning the difficult task of integrating the phylogenetically ancient Dominance/Submission Systems with the newer Attachment/CARE Systems. These systems accompany very different adaptive goals which can easily give rise to mutual conflict and can in turn make the balance of the BrainMind precarious and vulnerable to mental suffering.

## Introduction

Researches on *human development* (Boughner and Rolian, 2016) have progressively highlighted how the latter has become particularly complex during the evolution, compared to the development of other animal species. In this complexity, *adolescence* is an extremely important developmental stage, because of all changes occurring in the human *BrainMind* (Panksepp, 2011). *Adolescence* in humankind is characterized by multiple aspects—as will be discussed through this article—the most impressive of which is a significant restructuring of the brain (Arain et al., 2013; Walker et al., 2017) within a new hormonal context (Wierenga et al., 2018).

Studies of brain restructuring during *adolescence* have shown the emerging of *the dual systems model* (Pfeifer and Allen, 2012; Gladwin and Figner, 2014; Shulman et al., 2016), in which the limbic system—the seat of emotional functioning and reward—develops before the prefrontal cortex, involved in cognitive and regulatory processes (Padmanabhan et al., 2011; Casey et al., 2016). This model that traced back to *adolescence* the etiology of risk-seeking behaviors, the vulnerability to social frustrations and therefore the development of psychopathology, partially highlights the problem (Crone and Dahl, 2012). Highlighting the poorer regulation of cortical structures on the subcortical ones during *adolescence, the dual systems model* has contributed to paying less attention to the subcortical component. The latter has been relatively little studied as a set of motivational/emotional systems, biologically predetermined and expressing peculiar, endogenous intentionality. *Affective Neuroscience* (Panksepp, 1998) have contributed in the last two decades to reduce this imbalance, significantly bringing attention back to instinctual systems. These systems with peculiar neuronal and hormonal characteristics organize the adaptive behavior of mammals, including the human species, towards the material and relational environment.

What are these instinctual/emotional systems at the basis of mental and relational life? In *Affective Neuroscience*, Panksepp (1998), highlights the presence in mammals of several motivational/emotional systems responsible for regulating the interactions between conspecifics. Those are the SEEKING^1^ system that corresponds largely to the mesolimbic dopaminergic (ML DA) system, as will be discussed below; the LUST system that regulates sexuality, the PANIC/GRIEF system that regulates the interaction between caregiver and offspring and works together with the CARE system. In this article, we are going to use Attachment/CARE (Bowlby, 1969) to indicate the complex PANIC/GRIEF and CARE systems of Panksepp. Then there is the PLAY system that regulates the social interactions both in puppies and children and facilitates learning related to competitive interactions. Competitive interactions between sexually mature conspecifics are regulated by the *inter-male aggression*^2^ (Panksepp and Zellner, 2004; Kroes et al., 2006), also referred to as the urge for *social dominance* (Panksepp, 1998; Panksepp and Biven, 2012), Dominance system (van der Westhuizen and Solms, 2015) or *agonistic behavior* (Scott and Fredericson, 1951). The urge for *social dominance* is considered a “complex” secondary system made up of three primary emotional systems: “*Contributing factors (to social dominance n.d.r.) include* *SEEKING and RAGE, as well as FEAR, and surely early experience with the rough-and-tumble PLAY system are involved as well*.” (Panksepp and Biven, 2012, p. 169). Even if the RAGE system can be activated during the inter-male fighting, it is believed that the urge for *social dominance* has to be distinguished from that system (Panksepp and Biven, 2012, p. 169). The Dominance system “neurogeographically” includes the high density of testosterone receptors running from the medial amygdala, through the preoptic, anterior hypothalamic area, and down to the brainstem PAG (periaqueductal gray; Panksepp, 1998; Panksepp and Biven, 2012).

The urge for *social dominance* is in complementary interaction with the primary emotional system of FEAR, which activates submissive behaviors aimed at inhibiting aggression in the rival. It is, therefore, more appropriate to speak of a complex motivational/emotional system of Dominance/Submission (Giacolini and Sabatello, 2019); an exhaustive description of its functioning will be given later. The Dominance/Submission system—found in all vertebrates starting from fishes and reptiles up to mammals—motivates the acquisition of territorial, food and sexual resources (Lorenz, 1963; MacLean, 1990) through which *Sexual selection* takes place (Darwin, 1871).

The SEEKING system, the LUST system, the FEAR system and the RAGE system—present in all vertebrates—are the archaic motivational/emotional systems, while the PANIC/GRIEF system, the CARE system and the PLAY system—present only among mammals and some species of birds—are phylogenetically the most recent ones.

These motivational/emotional systems can be considered from the *Life history theory’s* perspective (Del Giudice et al., 2015; Knowles et al., 2019), significant articulations of the appearance of the developmental stage in various species. Research on *adolescence*, within the *Life history theory* and *Affective Neuroscience* point of view, can provide useful indications for the understanding and treatment of psychopathology, which makes its appearance in an elective way in this period of life and therefore also of the *addicted behavior*. It is precisely the emergence of psychopathology on the human development scene that can find an explanatory and treatment model, considering it as an effect of the peculiar features of a*dolescence—*a human-specific evolutionary stage (Bogin, 1994, 1997, 1999; Bogin and Smith, 1996; Robson and Wood, 2008; Hochberg, 2009; Hochberg and Belsky, 2013)—characterized by the restructuring of interactions between the subcortical primary motivational/emotional systems. As we shall see, adolescence constitutes a switch from the phylogenetically more recent motivational/emotional systems of Attachment and CARE (ontogenetically the first ones activated) to the phylogenetically more archaic ones of sexuality (LUST) and Dominance/Submission, which introduce the subject into the dynamics of *Sexual selection* (Darwin, 1871). Until puberty, the interactions between the subject in developmental age and adults are primarily regulated by the motivational/emotional systems of Attachment/CARE, although behaviors of Dominance/Submission are observed among puppies and children (Strayer and Strayer, 1978; Hawley, 1999, 2002; Hawley and Little, 1999; Hawley et al., 2002; Pellegrini and Long, 2002; Pellegrini et al., 2007). With sexual maturation, priority goes not only to the motivational/emotional system of sexuality (LUST) but simultaneously to that of Dominance/Submission. The human *BrainMind* (Panksepp and Biven, 2012) is thus subjected to the need to integrate motivational/emotional systems, each characterized by specific neurophysiological and hormonal characteristics such as to determine intentional and relational objectives that can enter into mutual conflict. Adolescence is a developmental stage in which the work of *integrating* these motivational/emotional systems becomes a priority. This work can expose the subject to the difficulty of integrating these systems, due to the transfer of attachment bonds from caregivers to sexual partners (Cassidy and Shaver, 2016). In adolescence, these attachment bonds no longer derive from the biological predisposition—active at birth—within the temporal windows of *imprinting* (Lorenz, 1937), but they are the result of both *Sexual selection* and the Dominance/Submission system. This new dynamic among primary and secondary emotional systems can create a predisposition to psychopathology, including the most age-specific types of addiction, as will be described in this article.

The urge for *social dominance* is present in both male and female children, as evidenced by the studies on *Adrenarche* (Maninger et al., 2009), the transspecies studies on the PLAY system (Panksepp et al., 1984, 1985) and the researches on *developmental psychology* (Hawley et al., 2002). In males, the Dominance system is characterized by a major *coercive social dominance strategy* while in females by a major *prosocial dominance strategy* (Hawley et al., 2002). With sexual maturity, the Dominance/Submission system is first of all characterized by the production of gonadal testosterone, produced in an extremely greater quantity in males than in females, in which the hormonal system is significantly characterized by estrogens and menstrual dynamics. In adolescence, the Dominance/Submission system acquires centrality in the regulation of social relationships, in line with the theory of *Sexual selection* (Darwin, 1871), exposing individuals to the consequences of the *social rank hierarchy* dynamics (Price et al., 2007), which can be conceptualized as a *continuum* between a richer relational world for dominant subjects and a poorer one for subordinate subjects (Nader and Czoty, 2005; Nader et al., 2012b; also see the interesting article on The Matthew Effect by Sloman and Dunham, 2004). With adolescence, the creation of relational bonds is now largely mediated by the secondary emotional system of Dominance/Submission, such as the conquest of a partner, of friends, of adults consideration, even if each relational bond will be simultaneously regulated by emotions of the Attachment/CARE systems. As we shall see, social stress from both *losses in social encounters* named *Social defeat* and from *social loss, such as the loss of a loved one* both share some same neurohormonal mechanisms (Panksepp et al., 2002, p. 111). *Social defeat* stress, which is activated when the subject is defeated during social interaction and involves the decrease or loss of social bonds, determines the release of CFR (corticotropin-releasing factor) which activates both the FEAR/Submission system and the PANIC/GRIEF systems (Panksepp and Biven, 2012, p. 340). The intra-sexual competition and the *Social defeat* stress exposes the subject to neurophysiological effects that can predispose not only to depression but also to addictions (Zellner et al., 2011). In this article, the interaction between the sexual hormones, the stress axis (HPA) and the dopaminergic system is considered above all in relation to males, both for a lower complexity of the *neurohormonal systems* involved than for females and for a lack of transspecies studies that consider the interactions between intra-sexual competition, *Social defeat* stress and addiction in female individuals (Hawley et al., 2008; Nader et al., 2012a).

## ML DA-Seeking System in Motivated (*Addicted*) Behaviors

Investigations on the neurobiological processes involved in the establishment of addictive behaviors and habits indicate a primary role of the mesolimbic dopaminergic (ML DA) system and associated areas and circuits (Wise and Bozarth, 1987; Di Chiara and Imperato, 1988; White, 1996; Alcaro et al., 2007). Indeed, the activation of this system provides the effect of reward and influences the reinforcement-learning processes, conditioning the incentive value attributed to the stimuli and the behaviors in both *classical* and *operant conditioning* (Robinson and Berridge, 2001, 2003, 2008; Salamone and Correa, 2002; Everitt and Robbins, 2005). The ML DA system thus plays a fundamental role in the acquisition of compulsive habits as well as in the continuous reactivation of such habits by conditioned stimuli.

However, from a neuro-ethological perspective which focuses on the instinctive and unconditioned factors that also influence learning (Lorenz, 1965; Panksepp, 1998), the ML DA system is considered to be the neurobiological substratum of the SEEKING system. This system is believed to have an intrinsic psycho-behavioral function that has evolved to motivate organisms to explore and to look for any kind of stimulus necessary for survival and reproduction (Ikemoto and Panksepp, 1999; Alcaro et al., 2007; Alcaro and Panksepp, 2011). Such emotional disposition constitutes the instinctive basis of all motivated behaviors during the appetitive phase of exploration and searches for distal stimuli and it is distinct from the second phase of consumption of a proximal stimulus (Wise and Bozarth, 1987; Berridge and Robinson, 1998; Salamone and Correa, 2012).

Expanding on the perspective of *Affective Neuroscience* (Panksepp, 1998; Alcaro and Panksepp, 2014), we proposed that addiction is generally characterized by a narrowing and tightening of the SEEKING disposition around certain compulsive memories that result in it being activated exclusively in specific contexts and channeled only through specific sequences of procedural activities. This may be due to some form of control exerted by stored memories in the superior cortical and limbic structures that interact with the activity of the subcortical centers and in particular of the ML DA-SEEKING system (Alcaro et al., 2007). Indeed, neurobiological research on animal dependency models has shown that the formation of compulsive habits is accompanied by a gradual functional reorganization of the brain from the molecular level to the system-circuit level (Nestler, 2002, 2014). At the neurochemical level, for example, a strengthening of glutamatergic synaptic connections has been observed in some nodal centers of *cortico-striatal circuits* (Pennartz et al., 2009), which would give rise to the well-known phenomenon of sensitization through which dependent memory complexes acquire a disproportionate power of activation of the ML DA-SEEKING system (see Steketee and Kalivas, 2011 for a summary on the subject).

In other words, there is a weakening of the ability of the ML DA-SEEKING system to express itself independently of the link with dynamic representations closed within the *superior procephalic circuits* and formed following repeated experiences of conditioning. Such loss of functional autonomy of the ML DA-SEEKING system constitutes, therefore, a common root for all forms of addiction or dependence, despite each having its basis in the action of specific memory complexes (Alcaro et al., 2007; Alcaro and Panksepp, 2011; Alcaro, 2019).

## ML DA-Seeking System in Adolescence

Although the evidence suggests that the loss of functional autonomy of the mesolimbic dopaminergic system is promoted and enhanced by specific experiences that progressively induce addiction, human and animal studies of dependence models have identified some neurobiological factors that can predispose an individual to develop compulsive behaviors. One of the most prominent of these factors has proved to be an endogenous predisposition to the dysregulation of the functioning of the ML DA-SEEKING system (George et al., 1995; Grace, 2000; Marinelli and White, 2000; Chefer et al., 2003; Alcaro et al., 2007; Alcaro and Panksepp, 2011). Such dysregulation causes the system to be hyper-reactive to particular environmental conditions characterized by the presence of novelty or artificial reinforcements such as substances of abuse (Piazza et al., 1989; Pierre and Vezina, 1997). Individuals characterized by this hyper-reactivity have been classified as “novelty seekers” or “sensation seekers” since they show strong attraction towards novel environments, sensorial stimulation, and high-risk settings (Bardo et al., 1996).

Interestingly, adolescents also show heightened orientation towards rewards in the environment including preferences for novelty, increased interest in risky situations, and other forms of emotional and sensorial stimulation (Wahlstrom et al., 2010). Following these observations, fMRI studies have shown that some neural systems innervated by the ML DA-SEEKING system are significantly more active in adolescents than children or adults when receiving primary rewards (e.g., sweet liquid; Galván and McGlennen, 2013), secondary rewards (e.g., money; Ernst et al., 2005; Galván et al., 2006; Van Leijenhorst et al., 2010), or social rewards (Guyer et al., 2006; Chein et al., 2011), as well as in the presence of appetitive social cues (Somerville et al., 2011). Such spikes in activity are associated with compromised cognitive control (Somerville et al., 2011) and increased self-reported risk-taking (Galván, 2014).

Accordingly, it has been hypothesized that the ML DA-SEEKING system is at a functional ceiling during adolescence (Chambers et al., 2003), as evidenced by overall higher tonic DA levels, peaks in DA cell firing, greater DA innervation, and increased DA receptor densities (see Padmanabhan et al., 2011; Padmanabhan and Luna, 2014). Therefore, the ML DA-SEEKING system is thought to be in a state of “overdrive” during adolescence, which appears to have important functional significance for behavioral outcomes and increases susceptibility to the onset of addiction and many other psychiatric disorders.

However, although the prevailing view is that heightened activity of the ML DA-SEEKING system represents a liability that orients adolescents towards risky behaviors and results in compromised well-being (e.g., Chambers et al., 2003; Casey et al., 2008) It has also been proposed that increases in risk-taking behaviors may also be adaptive for promoting survival and skill acquisition (Spear, 2000; see below).

Indeed, the overdrive of the ML DA-SEEKING system can be linked to the developmental phase of sexual maturation (see below) and to the explosion of hormonal and libidinal energy resulting in the search for contexts and situations in which it can be adequately satisfied (for a *neuro-psychoanalytic* point of view see Solms and Turnbull, 2002). Sexual drive (LUST system) is one of the main biological motivators capable of promoting and facilitating the activation of the SEEKING system (Panksepp and Biven, 2012). Moreover, sexual maturation affected during adolescence leads to a transition from infancy to adulthood, from the familiar environment to a complex and multiform social world (as we will see in detail below). From an *Affective Neuroscience* perspective, such change is also characterized by the transition from an emotional base cantered on the secureness of attachment (CARE disposition, PANIC disposition) and the joy of PLAY towards an emotional base cantered on the lust for sex and the competitive activity towards conspecifics (DOMINANCE, RAGE)^3^, although the urge for *social dominance* is also present among the puppies and children (see above).

In such a context, the tendency to explore and take risks may provide an adaptive function that affords a unique opportunity for adolescents to attain new experiences at a time when they are primed to learn from their environments and leave the safety of their caregivers (Spear, 2011). Therefore, we believe that the development of sexuality and the correlated ML DA-SEEKING, along with the competitive drive within the sexual selection, overdrive constitute the main vectors that lead the adolescent to abandon the safe haven and bonds of parental attachment and to embark on the difficult waters of an open and complex sea made up of uncertain, problematic and dangerous relationships and new unpredictable and uncertain contexts and situations. This journey is the necessary prerequisite for reaching adulthood with the consequent relocation of one’s individual identity from the family matrix to that of the shared social world, in which adolescents can find positive opportunities by exploring environments more suited to their genotype than those offered to their family matrix (Scarr and McCartney, 1983). In the next paragraphs, we will see this journey both from a phylogenetic and ontogenetic point of view.

Although adolescence is generally characterized by an ML DA-SEEKING overdrive, individual vulnerability to develop an addiction may be more specifically related to an increased electric responsivity of ML DA neurons to glutamatergic and other excitatory input (Marinelli and White, 2000; Alcaro et al., 2007). Such hyper-reactivity of the SEEKING system may be also related to a deficit in the tonic DA transmission, that is in the levels of DA molecules that extend outside the synaptic space and change slowly because they are relatively independent of nerve impulses (Grace, 2000; Chefer et al., 2003). Indeed, as a result of inhibitory regulatory processes on type D2 autoreceptors, the tonic levels of DA inhibit the readiness for the electrical discharge of DA neurons (Grace, 2000; Schmitz et al., 2003) and then tend to limit the tendency to search for environmental conditions characterized by novelty or artificial reinforcements such as drugs of abuse (Piazza et al., 1989; Pierre and Vezina, 1997). Therefore, the increased risk in adolescence may be related not only to an unspecific DA overload but instead to an enhanced ratio between the electric reactivity of ML DA neurons and the levels of tonic DA and D2 autoreceptors (Alcaro et al., 2007; Alcaro and Panksepp, 2011).

## Sexual Reproduction (Lust) and Competition

Sexual maturation determines in all vertebrate species, particularly mammals, the ability to reproduce and activates at the same time a process called *Sexual selection* by Darwin (1871).

Sexual reproduction also involves the drive to mate that is at the same time inextricably connected to the drive to compete with the individuals of the same species and gender (usually males) to gain access to the sexual reward.

This classic theory of motivational/emotional systems connected to the dynamics of *Sexual selection* has been expanded in recent years by studies that show that the urge for *social dominance—*is a strategy for *Sexual selection*, which is in itself a source of reward (Chester, 2017). Attacking a conspecific to impose one’s desire and dominance is an expression not only of the *urge for social dominance* (van der Westhuizen and Solms, 2015) but also a source of specific gratification (Chester, 2017).

*Inter-male* aggression in *Agonistic behavior* (Scott and Fredericson, 1951), highlights the complementary behavioral characteristics in combatants, such as agonistic engagement characterized by manifestations of aggressive threats and pacification, submission, avoidance behaviors in the defeated contender (see below). Unlike predatory aggression, intraspecific contests would be rarely fatal (see for a critic to this classic ethological observation Natarajan and Caramaschi, 2000), except in the human species where the evolution of the brain would have determined a specific use of aggression within the paradigm of *Sexual selection* (Eibl-Eibesfeldt, 1984). The hypothesis that aggression, within the paradigm of *Sexual selection*, may derive from predatory aggression—as we shall see below—seems to be borne out by the positive effect it evokes in the individual (Chester, 2017), *in contrast* to defensive aggression (see note 2) that generates a negative effect (Panksepp, 1998; Panksepp and Biven, 2012). Research by *Affective Neuroscience* also seems to indicate similar conclusions because: “[…] *the predatory aggression is the manifestation of the SEEKING urge*.” (Panksepp and Biven, 2012, p. 165) and: “[…] *the inter-male aggression that leads to dominance hierarchies seems to be an expression of the SEEKING system than of the RAGE system*.” (Panksepp and Biven, 2012, p. 168).

The positive effect associated with *inter-male aggression* is evident in the so-called PLAY system (Panksepp, 1998; Burgdorf et al., 2008; Graham and Burghardt, 2010; Panksepp and Biven, 2012), where both males and females playing tendencies are quite comparable (Panksepp, 1998, p. 230–281), even if some researchers highlighted that male infants tend to display more agonistic-like behaviors, while female infants display more social behaviors and behaviors reminiscent of mating (Graham and Burghardt, 2010).

The *inter-male* aggression related to positive affective experience has traditionally been linked to the pleasure of revenge, as retaliation for the violence suffered (Chester and DeWall, 2016). Neuroimaging studies have shown, during vengeful aggressive behavior, both the activation of areas of the *ventral striatum—*part of the ML DA system assigned to the reward—and the reduced activation of the *prefrontal lateral cortex* connected to its regulation (Chester and DeWall, 2016; Chester, 2017; Chester et al., 2019).

The activation of the dopaminergic system connected to aggression, however, is determined not only in aggression by retaliation but also in the absence of an avenging motivation such as proactive aggression or “appetitive aggression” (Carver and Harmon-Jones, 2009; Weierstall and Elbert, 2011; Hecker et al., 2012).

This aspect has been identified as being present in the so-called sensation-seeking personalities in which the pleasure determined by the elevation of the dopaminergic arousal is positively connected to experiencing a strong aggressive activation as an expression of predatory and inter-male aggression (Miller et al., 2012; Chester et al., 2016; Chester, 2017). The significant involvement of the ML DA-SEEKING system in the dynamics of *inter-male* aggression is evidenced by studies that have highlighted characteristics specific to addiction dynamics (Golden and Shaham, 2018). Animals that have experienced dominant aggression on a conspecific tend to prefer places where this experience has occurred and to repeat these behaviors on subordinate conspecifics (Legrand, 2013; Chester, 2017). Chester writes: “*This suggests that aggression is intrinsically reinforcing*” Chester (2017). It is necessary to add and underline an element which is not entirely evident in the studies cited, that this “appetitive aggression”: an expression of that particular aggressive motivational-emotional system which—as explained above—developed according to the *Sexual selection* (Lindenfors and Tullberg, 2011) and which is called *inter-male Agonistic behavior* (Scott and Fredericson, 1951), or *Dominance emotional system* (van der Westhuizen and Solms, 2015). To this latter denomination, we prefer the *Dominance/Submission* motivational/emotional system (Giacolini and Sabatello, 2019), because in our opinion it explicitly highlights the complex aspect of this *inter-male* aggressive system, which has the FEAR system as a complementary motivational system (Fish et al., 2005). In any species, the competitive contest does not involve the physical elimination of the contender (see above), as it happens in predatory aggression. This peculiarity was made possible by the function of the FEAR system within the competition, whose manifestations (of the FEAR system) in all species have the power to inhibit aggression in the victorious contender, preventing the elimination of the overwhelmed adversary (see van der Dennen, 2005 for review).

In social species, the FEAR system has given rise to the so-called *Submission* or *Pacification* behaviors, whose signaling is the basis for the formation of rank structures, allowing the coexistence of dominant and submissive individuals. These behaviors have also been called *Yielding subroutines* or *Involuntary Defeat Strategy* (Sloman and Gilbert, 2000; Sloman, 2002) to highlight the dimension of being forced or compelled present in the individual to maintain the rank structure. Such behaviors are considered analogous to those observed in humans manifesting depression (Gilbert, 1992, 2006).

On the other hand, dominant individuals manifest—in an equally compulsive way within the rank structure—*Dominance subroutines* or *Involuntary Dominant Strategy* (Sloman, 2002): the expression of the aggressive component of the motivational/emotional system of *Dominance/Submission* that has the function of reaffirming and verifying the supremacy of dominant individuals over subordinates. *Dominance subroutines* or *Involuntary Dominant Strategy* are considered analogous to the behaviors manifested along the manic spectrum (Sloman, 2002; Malatynska and Knapp, 2005; Johnson and Carver, 2012; Logan and McClung, 2016; Harrison et al., 2018) that are also characterized by the compulsive and unconscious dimension of these emotional states.

It can be assumed that these behaviors function to keep the hierarchies relatively stable and therefore the social structure of conspecific groups, thus favoring their synergy. If *inter-male* aggression—as expressed in *Dominance subroutines—*is a source of reward and potentially of addiction (Golden and Shaham, 2018), the FEAR system can likewise cause the activation of the dopaminergic system, opening the way to compulsive and addictive behaviors expressed as *Yielding subroutines* or *Involuntary Defeat Strategy*. It is now well established that the ML DA-SEEKING is activated not only in the presence of stimuli with a high reward content but also in the presence of adverse stimuli (Ikemoto and Panksepp, 1999; Alcaro et al., 2007). In the latter case, the ML DA-SEEKING appears to drive a search for “safety” (Ikemoto and Panksepp, 1999).

Consequently, the depletion of the dopaminergic system ML DA-SEEKING in the depressive state (Panksepp and Watt, 2011) occurs at the same time as its activation. This contradiction has recently been explained by discovering that the dopaminergic system is not homogeneous, but it is composed of various subsystems. These studies have highlighted how the ML DA system is formed of multiple subtypes of DA neurons with particular axonal projections and inputs, distinct anatomical, molecular and electrophysiological characteristics (Ikemoto, 2007; Lammel et al., 2012, 2014).

It appears that some DA subsystems are activated in the presence of reward stimuli, while others in the presence of stimuli with salience, whether positive or negative, but all subsystems transmit an *alerting signal* (Bromberg-Martin et al., 2010).

The ML DA-SEEKING System thus appears to be a powerful mechanism that can push the individual towards rewarding behaviors, such as aggression for dominance, but can also keep active a depressive emotional dimension connected to a subjective “belief of defeat,” which activates automatic protective submission behaviors to maintain a state of “safety.”

It should be noted, that the depression considered here is not the one resulting from separation (see the next paragraph) and therefore from the activation of the attachment system or PANIC/GRIEF system and the diminished brain reward/ML DA-SEEKING (Panksepp and Watt, 2011), but rather the effect of the dynamics of the Dominance/Submission motivational/emotional system. However, as will be highlighted later, the neurohormonal dynamic typical of separation will be “secondarily” activated in the defeated subject of the social competition.

Depression, as an emotional manifestation caused by the activation of the FEAR/Submission system (Gilbert, 1992, 2006) and by *Social defeat* (see below), will provide a means to achieve “safety” from the threatening aggression of a dominant and therefore from the source of a negative effect (see about the activation of VTA DA neurons in response to reward or aversive stimuli: Tanimoto et al., 2004; Brischoux et al., 2009; Lammel et al., 2014).

## From Vertebrates to Mammals, Towards *Homo Sapiens*

To understand the problem of addiction, a complex model that involves the interaction of the numerous biopsychosocial variables is required (Griffiths and Larkin, 2004; Lewis, 2005; Griffiths, 2008; Ratliff et al., 2016). Similarly, adolescence—as a topic of both research and mental care—involves considering the interaction between multiple factors of varying nature. During this developmental phase of a subject’s life, many factors are at work including the development of the soma, the maturation of various body parts, in particular the sexual and cerebral apparatus. Along with this biological development, the *adolescence* stage is characterized by the cultural dimension that is ritualized in multiform ways that lead to entry into adulthood (Kaplan and Garner, 2017; Kunnen et al., 2019).

The mind is the epiphenomenon of the functioning of the cerebral organ, which has evolved over millions of years in its effort to adapt to the environment, first natural and then relational (Alexander, 1990), constituting a complex *BrainMind* system (Cacioppo et al., 2014; Panksepp, 2014).

*Adolescence* is known to constitute a new phylogenetic developmental stage, added along with *childhood*, during the evolution of the genus *Homo*. This has allowed a better articulation of the stages already present in more evolved mammals, namely *infancy*, *juvenile*, and *sexual* *maturity*. One of the main causes of these stages of appearance, which have temporally extended the individual growth, is to be found in the massive increase of brain mass that characterizes the human species (Robson and Wood, 2008). The appearance of the *adolescence* growth stage, which extends the stage of puberty to a relatively long period, is assumed to be related to the further increase in the offspring survival rate. Thanks to the help given by young adolescents in raising siblings during *childhood*, the mother was allowed to engage in new pregnancies, contributing to the population growth (Bogin and Smith, 1996). Alongside this aspect connected to the aid in the rearing of offspring, elective for young adolescent females, there is another even more significant one: the learning function allowed by this new developmental stage, namely the learning of relational modalities in the social context, which were becoming more and more complex (Hochberg and Konner, 2020), and of the technological resources that were developing (Robson and Wood, 2008). Among the relational learning peculiar to the human species, a prominent place concerns the intra-gender competition that is *inter-male aggression*, elective in young adolescent males, and typical of *Sexual selection*. However, intra-sexual competition in the human species has been significantly complexed, implying not only the dimension of the conflict between individuals (as among other animal species), but also and above all the dimension concerning competition between groups (Van Vugt et al., 2007). The latter seems to be a peculiarity of the human species, partially shared by other evolved primates such as chimpanzees and orangutans (Wilson and Wrangham, 2003). Competition between groups indicates a complex regulation of the RAGE and FEAR motivational/emotional systems (Lebel, 2017), which has allowed the ability to tolerate proximity between conspecifics of the same sex, partially inhibiting the aggressive and distancing tendency. This has allowed cooperation to conquer the territorial resources, food, and sexual relations of another group of conspecifics. And precisely the ability to cooperate—specific to the human species (Puurtinen and Mappes, 2009)–would have found its origin and enhancement from intra-sexual group competition, as claimed by the *Male Warriors Hypothesis* (Van Vugt et al., 2007; McDonald et al., 2012). According to this theory, cooperation would be the effect of the male individual’s discovery that joining in intra-sexual competition determines a greater probability of victory, as well as survival both in obtaining food and in defending themselves from predators. The competition between groups would have selected a particular tendency to be afraid and attack those who do not belong to their group and to have instead positive effects towards members of their group—a propensity identified even among the highly evolved primates (Mahajan et al., 2011). Competition between groups (elective among males) would have contributed to determining the propensity of these males to remain within the group to which they belong at the period of mating, while the females, on the contrary, to migrate towards the husband’s group (Knipper et al., 2017). This greater propensity of males to feel part of a belonging group and to start a conflict with conspecifics of the outgroup is significantly correlated with testosterone production (Reimers and Diekhof, 2015; Muñoz-Reyes et al., 2020), which is extremely higher in males than in females. “[…] *humans, particularly men, may possess psychological mechanisms enabling them to form coalitions capable of planning, initiating and executing acts of aggression on members of outgroups (with the ultimate goal of acquiring or protecting reproductive resources)*” (McDonald et al., 2012, p. 671). Competition between groups, therefore, highlights the evolution of intra-sexual fighting that requires the individual to be able to combine the motivation for intra-sexual aggression and the fear of the opponent with the ability to create ties of proximity, to build functional alliances for struggling. This ability to compete through the group is an acquisition that seems to have been favored precisely by the onset of a long period of *adolescence*. The *initiation rites* (which took place during the period of adolescence; Alexander and Norbeck, 2011), sanctioned in the so-called primitive populations the transition from the indistinct identity of a *child* to that of an *adult* man. In particular, those related to the acquisition and learning of warrior skills, as well as tolerance and danger management. The *rites of passage* (Abeliovich, 2018) are, therefore, a window on the modeling of the primary and secondary motivational/emotional systems during adolescence, each characterized by a peculiar intentional organization that guides the behavior of single individuals (Montag and Panksepp, 2017). Cooperating to compete is an evolutionary acquisition of the great apes, whereby as in chimpanzees, cooperative behavior can be seen in group hunting, territorial defense, and alliances during male power struggles (Suchak et al., 2016; Massen et al., 2019). In adolescence, cooperating to compete in the new status makes the young adolescent feel inserted in a network of bonds with other peers and, at the same time, makes him perceive the ability to compete effectively. The emergence of psychopathology in adolescence, including substance use or addictive behaviors, highlights the adolescent’s dramatic belief that he is unable to combine the competitive drive with the cooperative drive and to transfer the attachment bonds into a new sexual relationship (Cassidy and Shaver, 2016). Cooperating to compete therefore implies the ability to tolerate the inevitable experience of *Social defeat*, implicit in the structuring of rank within the human group. The latter is an experience that makes the adolescent subject particularly vulnerable to mental pain, as the experience of Attachment-Care systems that had primarily organized his relational and mental life is still present in him.

Paraphrasing the zoologist Richard D. Alexander, the Environment of Evolutionary Adaptation—EEA (Bowlby, 1969) of *Homo sapiens* is no longer the material one but the relational one (Alexander, 1990). Being able to integrate motivational/emotional systems, characterized by purposes, intentionality, and therefore by extremely different *BrainMind* states, is the peculiar work of the adolescent period that makes the young subject vulnerable to psychopathology and addiction.

The *Affective Neuroscience* have (Panksepp, 1998) highlighted how the instinctual-emotional heritage is itself the primary form of consciousness through which human intentionality is expressed (Solms, 2019). Mental functioning cannot be understood, therefore, as a simple learning function, nor consciousness as a simple function of the corticalization of the human species. Based on research with animals (Panksepp, 1998) and humans (Panksepp, 2014; Solms, 2019), *Affective Neuroscience* has highlighted how the instinctual-emotional heritage is itself the primary form of awareness through which human intentionality is expressed.

This approach leads to questions about the phylogenetic heritage that led to the emergence of human psychic functioning and from this perspective the motivational/emotional systems are of particular importance. The latter are phylogenetically *homologous* in mammals and in the human species and they are wired into the subcortical structures between the brainstem and the limbic system, which are currently considered the roots from which the cortical, autonoetic and reflexive consciousness has evolved in a “bottom-up” and “top-down” mutual interaction (Panksepp and Solms, 2012; Solms, 2018).

## Attachment, *Infancy*, and Mental Pain

In this article, attention will be paid to the interaction between the “two complexes” motivational/emotional systems crucial for understanding *adolescence* and the dynamics of addictions: the Attachment/CARE systems and the Dominance/Submission systems (Giacolini and Sabatello, 2019).

The well-documented Attachment system (Bowlby, 1969) forms the basis of the child’s or offspring’s well-being when close to a reference adult or caregiver and involves a mental state that is largely determined by the release of *endogenous opioids* on *mu receptors* when offspring and caregivers are in proximity (Insel and Young, 2001).

The removal of the caregiver causes a depletion of these opioids at the receptor level and the implementation of *dynorphin production on k receptors* (Shippenberg et al., 2007), both of which cause a sense of discomfort and alarm in the offspring (Panksepp, 1998). This malaise occurs not only in infancy but can manifest itself at other times in life following separation from a significantly important individual (Cassidy and Shaver, 2016).

Both Spitz and Wolf (1946) and Bowlby (1969) have been accredited for their important contributions to the study of the separation of children from their caregivers. Spitz and Wolf (1946) has identified analogies between the stages of the prolonged separation process in young children (under 1 year of age) called the *Emotional Deprivation Syndrome*, which can lead to *Anaclitic depression* and the pathogenic effects of chronic stress referred to as *General Adaptation Syndrome* (stress syndrome) by Selye (1936) a few years earlier (Selye, 1936, 1946, 1956; see for current discussion Del Giudice et al., 2018). Here we anticipate the observation that drug withdrawal can be considered a *General Adaptation Syndrome* (Chartoff and Carlezon, 2015).

Descriptions of children’s reactions to separation from caregivers are marked by specific behavioral and neurophysiological states summarized in three phases related to the progression of separation. Bowlby (1969) defined the first phase as “Protest,” characterized by active recalls to the missing caregiver. If the separation continues and the child does not succeed in obtaining proximity to the sought-after parent, the phases of “Despair” and then of “Detachment” occur. These are identified by a gradual and progressive inhibition of the recall and search of the caregiver (Bowlby, 1969). These descriptions of the separation process identified by Spitz and Bowlby have been supported by the identification of a specific neuronal circuit that regulates the separation process, called the PANIC/GRIEF System (Panksepp, 1998). This neuronal pathway is composed of the *Anterior Cingulate, the Dorsomedial Thalamus, the Periaqueductal Gray*, and some regions of the *cerebellum*. The activity of other areas has been identified only in animals, but being so small, PET in humans hardly highlights them: *the ventral zone of the* *septum—Dorsal Preoptic Area—Bed Nucleus of Stria Terminalis—BN* (Panksepp, 1998).

The PANIC/GRIEF system is also characterized by specific neurochemistry that has a central point in the *endogenous opioid system*. The separation that gives rise to protest and discomfort is correlated with the depletion of endogenous opioids. If the separation continues, the effect of the continuing state of stress causes the prolonged alteration of the *HPA axis* (*Hypothalamic–Pituitary–Adrenal Axis*) and contributes to a hyperproduction of the *opioid dynorphin* (Bruchas et al., 2010).

This in turn, through the innumerable *kappa receptors* (Shippenberg et al., 2007) found in the ML DA system, inhibits both dopamine production in the *ventral tegmental area VTA* and its release in the *nucleus accumbens* (Watt and Panksepp, 2009).

The subsequent dopamine depletion is expressed as a depotentiation of the ML DA SEEKING system that governs the appetitive behavior (Panksepp, 1998; Watt and Panksepp, 2009; Zellner et al., 2011).

The joint effect of these two depletions characterizes not only the dynamics of separation from a caregiver but also those of depression in adulthood and the abstinence both from substance and non-substance addictive behaviors.

Why this particular interest in the Attachment (PANIC/GRIEF system) and in particular for the dynamics connected to separation? Because it is probably the biggest cause of our mental pain (Panksepp and Biven, 2012). The recognition of the caregiver consolidated during *infancy*, brings with it the stabilization of the need for his/her presence, whose absence will activate the stress and depletion processes mentioned above, responsible for mental pain and analogous to a real *craving* (uncontrollable compulsive desire; Zellner et al., 2011).

From this point of view, the attachment bond can be considered as the prototype of the “first addiction” (Zellner et al., 2011). In this regard, as pointed out by Jaak Panksepp (Yovell et al., 2016), opiates are the first identified antidepressant drugs, bearing in mind all the inevitable problems they entail.

Opiates use is known to reduce and replace the need for social relationships. However, soon the substance that creates this social well-being, heroin for example, inevitably leads to the learning of a reinforcement that activates the appetitive system to seek it out in a paroxysmal way.

Thus attention shifts to the ML DA SEEKING system. Any addictive substance, alcohol, tobacco, cocaine, et cetera, activate the increase of extracellular dopamine concentration in the dopaminergic system sites and especially in the *nucleus accumbens* (Volkow and Li, 2004). Quickly, the ML DA SEEKING system becomes the driving center of addictions, both for substance and non-substance addictive behaviors (like gambling, internet, et cetera) that have the same power to subvert the system (Koob and Volkow, 2016; Uhl et al., 2019).

## From Infancy to Juvenile Age

In most mammals, there are only two stages of development: *infancy* and *sexual* *maturity*. The transition from one to the other is quite sudden and puberty occurs when growth rates are decreasing (Bogin and Smith, 1996). In more social species—such as wolves, lions, elephants, and primates—a third stage has been added after *infancy* (namely after weaning): the *juvenile* stage (Bogin and Smith, 1996), which has postponed the advent of puberty. *Infancy* indicates the period of maximum dependence of the puppy from the *caregiver*, represented electively by breastfeeding. In the human species—as mentioned above—it is replaced by *childhood* around the age of 3. *Childhood* (3–6 years old) is a period in which the immaturity of the dentition and digestive tract requires food prepared by the caregiver, easily digestible, and very energetic to meet the nutritional requirements for the body growth, especially the brain. After *childhood*, the *juvenile* age takes over from around 6–7 years up to about 11–12 years. The *juvenile* stage is characterized by individuals sufficiently autonomous from their caregivers and with motor and cognitive resources such as to be able to provide in part to their sustenance and protection from predators (Bogin and Smith, 1996), even if they still maintain an interaction of dependence with the adult of reference. One of the characteristics of the *juvenile* stage, in some primates and in the human species, is the phenomenon of *Adrenarche* (Maninger et al., 2009). Around the age of 6–8, the maturation of the *hypothalamic-pituitary-adrenal axis* (HPA) is considered the beginning of the pubertal maturation process (Mundy et al., 2015). The maturation of the *adrenal cortex*, *the zona reticularis*, determines the production of androgens such as *dehydroepiandrosterone* (DHEA) and its sulfate DHEA-S along with androstenedione (A4) converted into a certain amount of *testosterone* (Antoniou-Tsigkos et al., 2019). The latter two, with the maturation of the *hypothalamic-pituitary-gonadal axis* (HPG), will be produced in greater quantities by the gonads, characterizing the actual sexual development of puberty. *Adrenarche* is therefore considered to be the beginning of puberty, or as it is defined as “adrenal puberty” (Del Giudice et al., 2009) before the visible signs highlight gonadal puberty. Adrenal androgens are neurosteroids active in brain regions involved in emotional and behavioral regulation (Maninger et al., 2009; Mundy et al., 2015). “Adrenal puberty” determines the appearance of physical signs such as pubic hair, the change in the composition of sweat that produces body odor, but the most significant changes are the behavioral ones shaped by *sexual selection* and related to competition within gender identity (Del Giudice et al., 2009, p. 2). The effects attributed to the *Adrenarche* correspond to those of the PLAY system described by the *Affective Neuroscience* (Panksepp et al., 1984, 1985; Panksepp, 1998). This motivational/emotional system is activated during the *juvenile* stage of mammals and is characterized by an intra-sexual competitive-like interaction, with the characteristic of reversibility (Panksepp and Biven, 2012). In fact, in these interactions regulated by the PLAY system, the competition does not definitively sanction a winner and a loser, as in the competitive interactions that will follow sexual maturation. This reversibility allows the contenders to maintain a mutually friendly interaction and a source of positive effects (Knutson et al., 2002). One of the evolutionary function of the PLAY system seems to be, therefore, to train juvenile subjects to assume behaviors that will be characteristic of adolescence and then of intra-sexual competition in adulthood, even if there is still no clear evidence of a significant continuity from the *rough-and-tumble* Play to mature *Inter-male* aggression in the executive brain mechanisms (Panksepp, 1998, p. 286). The subsequent and massive production of gonadal testosterone in puberty will determine the reduction of the PLAY System manifestations because the androgen hormone promotes aggression, especially between sexual adult male, leading animals and humans to real fights (Panksepp, 1998, p. 286; Panksepp and Biven, 2012, p. 362).

It is thus possible to hypothesize that the PLAY system—functionally connected to the *Adrenarche—*constitutes a developmental stage through which two motivational systems start to be mutually conjugated: the Attachment/CARE system and Dominance/Submission system. *Adrenarche*, through the production of adrenal hormones with a low androgen content, highlights their strategic function in activating in a reduced way the competitive behavioral system of Dominance/Submission. It can be assumed that this allowed the beginning of integration between these latter emotional systems and the Attachment/CARE motivational/emotional systems, making possible friendly proximity between the offspring while they are struggling.

## Social Competition in Human Development

While the motivational/emotional systems of Attachment/CARE have as a priority the maintenance of proximity between individuals (Cassidy and Shaver, 2016), the motivational/emotional systems of Dominance/Submission that regulate intra-sexual competition are predisposed to create distance between individuals to achieve an optimal dislocation of the population concerning environmental resources (Lorenz, 1963).

With puberty, the biological beginning of adolescence, the need to stay close to a caregiver is increasingly replaced by the need to join a peer group. Even before puberty, interaction with peers is fundamental for the proper functioning of mental processes (Pellis and Pellis, 2007; Vicedo, 2010), as considered above. With the beginning of adolescence the urge for *social dominance*, which already had its appearance with the *Adrenarche* (Del Giudice et al., 2009), now acquires particular importance (Weisfeld, 1999; Hawley, 2011), as a tool of sexual selection and to drive the adolescent away from the family environment (Weisfeldt and Woodward, 2004).

During adolescence, gonadal maturation increases the production of testosterone especially in males, with a ratio about ten times higher than in females (Braunstein, 2007), amplifying the *urge for* *social dominance*, which replaces Attachment as the main organizer of the relational mental life. The maturation of the sexual organs with the increasing production of testosterone, with related gender differences (Holder and Blaustein, 2013), stimulate the dopaminergic system functioning (Wahlstrom et al., 2010) connected to the production levels of the androgen hormone (Jardí et al., 2018). This may be one of the variables that determine greater competitiveness among males along with a stronger drive to seek out risky situations. It is believed to be also one of the causes of schizophrenic syndromes that are more prevalent in adolescent males (Trotman et al., 2013; Bratek et al., 2015), than females, while the latter display an increased incidence of depression (Sundquist et al., 2004; Sinclair et al., 2014).

Brain regions are not affected uniformly by the developmental effects of puberty. In particular, the subcortical areas of the ML DA-SEEKING system mature before the cortical ones (Wahlstrom et al., 2010; see above the *Dual System Theory*), predisposing the subject to both impulsiveness and addiction. In parallel, there is a proliferation of *glucocorticoid receptors* (GR) in the dopaminergic system, especially in *prefrontal cortical areas* (Sinclair et al., 2014). The latter is one of the main causes of vulnerability to social stress in adolescents.

Therefore, adolescents are more inclined to seek stimulation (Steinberg, 2007)—in particular social stimuli—compared to individuals in other stages of life, but at the same time, they are particularly vulnerable to social stress. This concept refers to the subject’s vulnerability to easily perceive himself as a loser compared to other conspecifics (Kroes et al., 2006) and therefore inadequate in the context of relationships. This results in negative self-evaluation and evokes a similar emotional state to the one generated by separation related to the Attachment system, because the *Social defeat*, *the loss of social status* and *the social loss*, *the* *loss of a loved one*, share some of the same neurohormonal mechanisms: *“Both types of social losses* *share certain* *key physiological features, such as arousal of the hypothalamic-pituitary-adrenal axis, and activation of non-specific arousals/attentional circuits like ascending norepinephrine and acetylcholine systems.”* (Panksepp et al., 2002, p. 111). This connection between *Social defeat* and social loss has been well demonstrated by the *resident-intruder* *paradigms*, according to which after defeat, if the loser is reunited with other “friendly” animals it recovers from the stress of defeat. On the contrary, if the loser remains isolated it shows signs of weight loss, heart rate disruptions, body temperature regulation, and increased fear (Panksepp et al., 2002) that characterize depression in the human species. This neuro-hormonal dynamic is activated not only as a consequence of competitive interactions in progress but also in the anticipation that they may happen: “*A psychosocial stressor is the anticipation, justified or not, that a challenge to homeostasis looms*.” (Sapolsky, 2005, p. 648).

The experience of *Social defeat* can be related to acute and circumscribed social stress or to a lasting state that then becomes chronic stress (Luckett et al., 2012; Gray et al., 2015).

Experiences of continuous *Social defeat*, such as causing chronic stress, have a significant effect on the dopaminergic system, reducing functionality (McLaughlin et al., 2006; Selten and Cantor-Graae, 2007; Selten et al., 2013). In animal models, *Social defeat* stress predisposes the individual to exaggerate subsequent addiction-like behaviors for cocaine, methamphetamine, alcohol, and opioids (Shimamoto, 2018). Those behaviors are related to the intensity, duration, frequency, and stress in defeated animals (Shimamoto, 2018). Exposure to adverse stimuli such as *Social defeat*, as well as positive reinforcement stimuli, increases concentrations of extracellular dopamine in the terminal areas of the ML DA system, nucleus accumbens, striatum, and prefrontal cortex (Tidey and Miczek, 1996; Piazza and Le Moal, 1998; Sapolsky, 2017). In defeated animals, during the social threat, extracellular dopamine levels in the accumbens and prefrontal cortex increase by approximately 160% from baseline (Tidey and Miczek, 1996).

The Dominance system is influenced by two hormones, testosterone and arginine-vasopressin (AVP; or ADH *antidiuretic hormone*). Both are involved in sexual dynamics (Panksepp and Biven, 2012), and the latter also in fostering social ties (van der Westhuizen and Solms, 2015). One of the effects of androgenic hormones is to block the separation alarm (or inhibit the PANIC/GRIEF system) and the social withdrawal in adult males (Enter et al., 2014).

As mentioned above, the Dominance system is complementary to the FEAR/Submission system. The latter is marked by the activation of the stress axis through the release of *corticotrophin-releasing factor* (CFR) and the production of *cortisol*, the neuro-chemical signature that characterizes the experience of *Social defeat* (Panksepp, 1998; Sapolsky, 2004, 2005). *Cortisol* acts by inhibiting the production and functioning of *testosterone* that promote Dominance behaviors and in turn, it reduces the blocking of the social separation alarm and the withdrawal (Enter et al., 2014; see above also Panksepp et al., 2002). At the same time, *cortisol* activates the production of the *opioid dynorphin* (already considered for the effects of separation) that depresses the ML DA system (McLaughlin et al., 2006). It is now well established that the factor that differentiates individuals in the dynamics of Dominance/Submission is the ratio between the basal level of *testosterone* and that of *cortisol* (Terburg et al., 2009; van der Westhuizen and Solms, 2015; Barel et al., 2017). Therefore, consequently, it can be hypothesized that this is one of the causes of the variability in addiction vulnerability during adolescence.

The effects of *cortisol* are, in turn, significantly inhibited by the release of endogenous opioids on *mu receptors*, or artificially by the administration of morphine as widely studied in “playful” interactions between males of various animal species (Panksepp et al., 1985; Panksepp and Biven, 2012). The administration of opioids in animals makes individuals dominant in “playful” competitive situations (Panksepp et al., 1985). This neurochemical dynamic highlights the close analogy and connection between competition stress and separation stress (Giacolini, 2019).

There is therefore a close correlation between the stress of *Social defeat* with the FEAR defensive system (Panksepp, 1998) and with the more phylogenetically recent PANIC/GRIEF emotional system. Both of these primary emotional systems share overlapping neuroanatomy, most notably the PAG (periaqueductal gray), and chemicals such as the neurotransmitters GABA, norepinephrine, serotonin, and dopamine. Furthermore, both are activated by CRF (corticotropin-releasing factor; Panksepp, 1998, p. 268; Panksepp and Biven, 2012, p. 334–340).

The positive correlation observed between secure attachment and social dominance and between insecure attachment and tendency to anxiety and behaviors of Submission and *Social defeat* (Irons and Gilbert, 2005; Hawley et al., 2008) is completely in agreement with the arguments presented above.

How do adolescence, attachment/separation, Dominance/Submission, *Social defeat*, and the dynamics of addictions combine? As described above, chronic stress has a negative effect on the ML DA system, altering its functionality and rhythmicity, and resulting in a significant reduction of D2 receptors. This reduction contributes to creating a predisposition in the individual towards substance or behavioral addictions, so by stimulating the production of dopamine—as mentioned above—the emotional experience (feeling) of well-being is induced. This experience then drives the subject to search for the object that has caused this emotional state to maintain it at an optimal level (Ikemoto and Panksepp, 1999).

In this regard, the study conducted by Morgan et al. (2002) is of particular importance because it is one of the few studies of animal models using primates (*Macaca fascicularis*), rather than rats or hamsters. In this study, adult male macaques subjected to a long period of isolation showed a depletion of D2 receptors in the *basal ganglia*, as observed in subjects with substance addiction or in victims of chronic stress. This depletion of D2 receptors can be closely linked to the vulnerability that subjects display after taking substances of abuse and towards which they subsequently become more easily addicted. The adult male macaques thus treated were in due course divided into groups and after about 3 months they could ingest cocaine. The substance was consumed by all the subjects but was subsequently sought compulsively by those animals belonging to the subordinate rank. The dominants were, on the contrary, resilient to addiction, because they had experienced the substance without developing an addiction. PET scans revealed that the addicted, subordinate animals continued to show depletion of D2 receptors in the region of the *basal ganglia* similar to the one following the period of isolation. The resilient and dominant animals showed instead a recovery to a level similar to the one they had before isolation. According to the authors, this experiment shows that in selected individuals with very similar phenotypic characteristics (basal levels of testosterone and cortisol analogs, et cetera), vulnerability to addiction may be determined by social position or rank that exposes submissive subjects to chronic stress and dominant subjects to the opposite effect (Sapolsky, 2004, 2005). Bearing in mind the questions that arise from extrapolating conclusions about human behavior based on animal models results, Morgan’s study is part of a broad line of research on the effects of *Social Defeat*, especially in adolescence, which indicates how it predisposes individuals to a vulnerability towards substance abuse and behavioral addictions (Sullivan et al., 2006; Tharp-Taylor et al., 2009; Topper et al., 2011; Shimamoto, 2018). It is worth mentioning that Björkqvist (2001), notes that the terms “dominant” and “subordinate” are used in animal studies, while the terms “bullies” and “victims” are used in human studies, in which the interaction between peers, students or colleagues are examined. *Social defeat* related to academic, work, and social status frustration has received less attention, although there are some important studies (Selten et al., 2013).

Comparative psychopathology has shown that subordinates are not only constantly in a state of stress, but they are also in a peripheral position concerning the group and more exposed to dangers (Sapolsky, 2004, 2005; Huhman, 2006).

Therefore, *Social defeat* exposes individuals to emotions (feelings) very similar to the experiences of separation. On the opposite, the achievement of a dominant place evokes feelings that can be considered analogous to that of reunification in the dynamic of separation from a caregiver (Kozorovitskiy and Gould, 2004).

## Conclusion

We have described above how sexual maturation contributes to the enhancement of the ML DA system that, in combination with hormonal changes, creates a powerful drive for a wider exploration of the world and sexual competition in search of an expected reward (Ikemoto, 2007).

But simultaneously, the ML DA system in adolescence also creates an equally powerful drive to avoid distress and to seek “safety” from social pain (namely *Social defeat*; Ikemoto and Panksepp, 1999).

In both cases, adolescence appears to be a period in which the human *BrainMind* is physiologically in a state predisposed to seek greater arousal (Chambers et al., 2003; Crews et al., 2007) or to avoid sources of pain through fixing on easily accessible sources of pleasure that mitigate or nullify the feared sense of threat (García-Oliva and Piqueras, 2016).

In adolescence, the dynamics of the Dominance/Submission system and its interaction with the Attachment/CARE system acquires a particular intensity due to the asymmetry in the maturation of the subcortical areas compared to the cortical ones (see above *the dual systems model*) which makes the subjects more exposed to the neuro-hormonal and psychological dynamics of these emotional systems, exposing teenagers to be particularly vulnerable to mental suffering and addictions.

The Attachment motivation system has long been acquired as an instinctual system that regulates relations between children and caregivers (Cassidy and Shaver, 2016). More difficult is the recognition that interactions and relationships between individuals can be equally regulated by the urge for *social dominance*. If sexuality has always represented the persistence of an instinctual heritage in human mental functioning (Freud, 1905), the competitive interaction—which in every culture influences the dynamics of social relations—is still little studied within the human brain. Adolescence, in this sense, is a phase of development particularly suited to study these instinctual dynamics and the difficult and conflicting work of integrating very different phylogenetically motivational/emotional systems. It is precisely the work of integrating these instinctual systems that creates a state of potential vulnerability to mental pain and consequent addicted problems related to it, together with the dramatic observation of the close functional interaction between the cultural dimension and motivational systems and their regulation (Kunnen, 2012).

In traditional cultures, the difficult passage characteristic of the adolescence stage has been ritualized through social practices that often consisted of removing adolescents from the family context and making them live together. During this transition phase, especially adolescent males were often subjected to tests of courage to prove their worth and their ability to survive by themselves. Furthermore, the rituals of initiation into adulthood often included the use of drugs and other practices that caused altered states of consciousness to lead the individual towards new forms of representation of reality. Such practices have been maintained, albeit in a modified form even in contemporary society. Many of the risk-taking behaviors typical of today’s adolescents can be seen as similar initiatory rituals which, however, very often have lost their socio-cultural reference and meaning and which therefore threaten to trap the individual in a spiral of compulsive behaviors with no way out. Moreover, the consequences of risk-taking are likely to be context-dependent. In our modern society, the environments where adolescents take risks (e.g., driving cars) may result in maladaptive instead of adaptive outcomes (Spear, 2008).

Within the *BrainMind* (Panksepp and Biven, 2012), the regulation and the integration between motivational/emotional systems are a central aspect in the construction of both normal and pathological personality functioning (Montag and Panksepp, 2017). However, as contemporary authors pointed out, the heuristic potential connected to the study of motivational/emotional systems has still to be widely explored (Maze, 1993; Boag, 2014), with particular regard to the urge for *social dominance* (Hawley, 2002; Johnson et al., 2012; Panksepp and Biven, 2012).

A limit of this article is that the authors do not address the contrast between the primary process and the secondary and tertiary processes (Panksepp and Biven, 2012) because it is beyond the scope of this article. We intend to address this issue in a future article.

## Author Contributions

The article was a collaborative, joint effort in which TG took the theoretical lead, AA introduced important neuroscientific contributions, DC played a more supportive and advisory role. All authors contributed to the article and approved the submitted version.

## Conflict of Interest

The authors declare that the research was conducted in the absence of any commercial or financial relationships that could be construed as a potential conflict of interest.

## Footnotes

^1^The capital letters used for motivational/emotional systems are a convention in Panksepp’s works both for labeling neurologically and for a need of a specialized terminology ones.

^2^“*Three distinct aggressive circuit hae been provisionally identified in the mammalia brain: predatory, intermale, and affective attack or RAGE circuits”* (Panksepp, 1998, p. 188).

^3^Although they are separate developmental phases, there are nevertheless interesting overlaps between the attachment processes and the processes of social competitiveness since some psychobiological indices of secure attachment are also common to individuals who show a propensity for dominance. As a single example, it is sufficient to note that the quantity of D2 receptors, which we can generally associate with the steady functioning of the ML DA-SEEKING system is a good indicator of secure attachment and social dominance. Conversely, lower concentrations of D2 receptors in striatal areas is an indicator of social submission and anxious insecurity that predisposes to the development and maintenance of compulsive behaviors (Morgan et al., 2002).
